# Cacao (*Theobroma cacao* L.) Response to Water Stress: Physiological Characterization and Antioxidant Gene Expression Profiling in Commercial Clones

**DOI:** 10.3389/fpls.2021.700855

**Published:** 2021-09-06

**Authors:** Mayra Andreina Osorio Zambrano, Darwin Alexander Castillo, Loyla Rodríguez Pérez, Wilson Terán

**Affiliations:** Plant and Crop Biology, Department of Biology, Pontificia Universidad Javeriana, Bogotá, Colombia

**Keywords:** drought, water deficit, chlorophyll fluorescence, gas exchange, photosynthesis, oxidative stress, water potential, RT-qPCR

## Abstract

The increase in events associated with drought constraints plant growth and crop performance. Cacao (*Theobroma cacao* L.) is sensitive to water deficit stress (DS), which limits productivity. The aim of this research was to characterise the response of seven (CCN51, FEAR5, ICS1, ICS60, ICS95, EET8, and TSH565) commercially important cacao clones to severe and temporal water deficit stress. Ten-month-old cacao trees were submitted to two treatments: well-watered and water-stressed until the leaf water potential (*Ψ*_leaf_) reached values between −3.0 and −3.5 MPa. The effects of hydric stress on water relations, gas exchange, photochemical activity, membrane integrity and oxidative stress-related gene expression were evaluated. All clones showed decreases in *Ψ*_leaf_, but TSH565 had a higher capacity to maintain water homeostasis in leaves. An initial response phase consisted of stomatal closure, a general mechanism to limit water loss: as a consequence, the photosynthetic rate dropped by approximately 98% on average. In some clones, the photosynthetic rate reached negative values at the maximum stress level, evidencing photorespiration and was confirmed by increased intracellular CO_2_. A second and photosynthetically limited phase was characterized by a drop in PSII quantum efficiency, which affected all clones. On average, all clones were able to recover after 4 days of rewatering. Water deficit triggered oxidative stress at the early phase, as evidenced by the upregulation of oxidative stress markers and genes encoding ROS scavenging enzymes. The effects of water deficit stress on energy metabolism were deduced given the upregulation of fermentative enzyme-coding genes. Altogether, our results suggest that the EET8 clone was the highest performing under water deficit while the ICS-60 clone was more susceptible to water stress. Importantly, the activation of the antioxidant system and PSII repair mechanism seem to play key roles in the observed differences in tolerance to water deficit stress among clones.

## Introduction

Cacao (*Theobroma cacao* L.) is a fruited tree native to the Amazon basin of South America (Motamayor et al., 2008; Cornejo et al., 2018), and its cultivation represents the main economic livelihood for smallholder farmers and landowners in several producing countries in Africa, Central America, and South America (Phillips-Mora et al., 2007; Baligar et al., 2008). Cacao beans are the main raw material of interest for the chocolate confectionary industry, and they are also used in the cosmetic or pharmaceutical industry, with increasing interest in recent years as a source of bioactive compounds (Prabhakaran Nair, 2010; Romero et al., 2017). Cacao bean production reached 4.6 million tons in 2017/2018 (ICCO, 2020), which benefited approximately 40 to 50 million people (Carr and Lockwood, 2011; Voora et al., 2019) and the global market is growing predicted to increase 7.3% from 2019 to 2025 (Voora et al., 2019). However, some important threats related to global warming may reduce this growth prediction during incoming decades, such as the higher incidence of water deficit and drought periods in current cacao cultivation areas (Läderach et al., 2013; WCF (World Cocoa Foundation), 2014; Schroth et al., 2016; Medina and Laliberte, 2017; Farrell et al., 2018; Gateau-Rey et al., 2018; Lahive et al., 2019; Hebbar et al., 2020). The development and productivity of cacao trees are affected by drought (Almeida and Valle, 2007). Soil water deficit may result from a reduced water inputs to agroecosystems (Gilbert and Medina, 2016), poor irrigation, high or low temperatures, or excessive application of mineral salts and fertilizers to soil (Tardieu, 2013; Basu et al., 2016; Qaderi et al., 2019). During water deficit, the transpiration rate exceeds water absorption by the roots, thereby reducing the water content in tissues and affecting both nutrient uptake and photosynthesis (Ahuja et al., 2010; Osakabe et al., 2014; Demidchik, 2015).

To cope with water deficit periods, plants have developed several strategies and response mechanisms that vary from one plant species to another and depend on the plant phenological state or the duration and intensity of stress (Nishiyama et al., 2011; Ara et al., 2013). Some of these strategies can confer tolerance to overcome water deficit and improve recovery after rehydration (Griffiths and Parry, 2002), which is a trait of high importance for plant breeding. By 2050, half of the currently cultivated lands will likely suffer from drought (Dai, 2011; Farnese et al., 2016). This acclimation response has a genetic base and involves all different levels of plant biology, e.g., epigenetic, molecular, biochemical, and physiological, in an integrated fashion. Importantly, crosstalk between signal transduction pathways and interplay between secondary messengers have been reported as key for the effectiveness of this acclimation response at a systemic level, where abscisic acid (ABA) is considered the chief orchestrator. As water deficit stress leads to oxidative stress due to photorespiration and a concomitant increase in ROS production, deleterious oxidative damage to proteins, lipids, photosynthetic apparatus and nucleic acids accumulates in cell tissues (Noctor et al., 2002). Therefore, ROS detoxification by enzymatic and nonenzymatic systems is a crucial and well-described mechanism developed by plants against drought-induced oxidative damage (Shao et al., 2008; Foyer and Shigeoka, 2011; Laxa et al., 2019). In addition, high activity levels of antioxidant enzymes, such as superoxide dismutase (SOD), NADPH oxidases (RBOH), glutathione S-transferase (GST), and alcohol dehydrogenase (ADH), have been shown to contribute to the attenuation of oxidative stress caused by water scarcity (Mittler, 2002; Ahuja et al., 2010) and can be used as indicators of tolerance or susceptibility to this abiotic stress (Saleh and Plieth, 2009).

The above effects of drought on plant fitness, as well as the different levels of response and mechanisms developed by plants, have been widely studied in the model plant *Arabidopsis thaliana* and in crops of world economic importance, such as rice (*Oryza sativa* L.) or maize (*Zea mays* L.) (Jackson et al., 2009; Akhtar and Nazir, 2013; Nakashima et al., 2014). However, this is not the case for many tropical crops of commercial importance, such as cacao. Few studies have addressed the important physiological effects of water deficit in cacao, which leads to abscission of vegetative and reproductive organs, a reduction in photosynthesis, water use efficiency and dry matter accumulation, and decreases cacao beans production yields by 10 to 89% (Zuidema et al., 2005; Köhler et al., 2010; Moser et al., 2010; Schwendenmann et al., 2010; Gateau-Rey et al., 2018). However, additional efforts are needed to increase our understanding of the different mechanisms supporting the cacao acclimation response to water deficit and its variability among genotypes (Ahuja et al., 2010; Osakabe et al., 2014; Farnese et al., 2016; Mosa et al., 2017). With this focus, we assessed the effect of a period of water deficit and recovery on the physiological response and behavior of seven cacao clones of commercial importance. Further expression profiling of genes involved in the oxidative response and soil water deficit was carried out on three clones representative of the different responses found.

## Materials and Methods

### Plant Material and Stress Treatment

This study was carried out at Bambusa Station (Geoambiente SAS), which is located in the dry premontane forest at 5°07′50″ north and 74°09′30″ west and 1,304 m.a.s.l. in Pacho (Cundinamarca-Colombia). All clones were propagated in a glasshouse by grafting and using Caucasia (Colombian) rootstock (Geoambiente SAS) for mass production. Ten-month-old well-developed seedlings were used for the experiment. The commercial clones EET8, FEAR5, CCN51, TSH565, ICS1, ICS60, and ICS95 were selected because they belong to the group of the most recommended materials for planting and marketing in Colombia by the National Cacao Council (Cardona, 2010) and based on their agronomic performance (Table 1).

**TABLE 1:** Cacao commercial clones used in this study.

Genotype (Nomenclature)	Identity	Genetic group	Origin	Agronomic traits	References
CNN51	Castro Naranjal Collection	Amazonian- Trinitarian Hybrid	Ecuador	High Productivity, early fructification, and diseases resistant	Boza et al., 2014; García Lozano, 2014; Perea Villamil et al., 2017
EET8	Tropical Experimental Station	Amazonian type-Trinitarian	Ecuador	High bean index	Joly and Hahn, 1989; Perea Villamil et al., 2017
FEAR5	Fedecacao Arauquita 5 RC ICA 4179	Trinitarian Hybrid	Arauquita (Arauca Colombia)	High quality and yield	Perea Villamil et al., 2017
ICS1	Imperial College Selection	Trinitarian Hybrid	Trinidad, Nicaragua, and Venezuela	High bean and pod index	Johnson et al., 2009; Perea Villamil et al., 2017
ICS60	Imperial College Selection	Trinitarian Hybrid	Trinidad, Nicaragua, and Venezuela	High bean and pod index, medium tolerance to moniliasis and witch broom diseases	Bekele et al., 2006; Johnson et al., 2009; García Lozano, 2014; Perea Villamil et al., 2017
ICS95	Imperial College Selection	Trinitarian Hybrid	Trinidad, Nicaragua, and Venezuela	High bean and pod index, medium tolerance to moniliasis and witch broom diseases	Bekele et al., 2006; Johnson et al., 2009; García Lozano, 2014; García Lozano and Moreno Fonseca, 2016; Perea Villamil et al., 2017
TSH565	Trinidad Selection Hybrid	Trinitarian	Trinidad	High production and moniliasis resistance	Johnson et al., 2009; Perea Villamil et al., 2017

**FIGURE 1:**
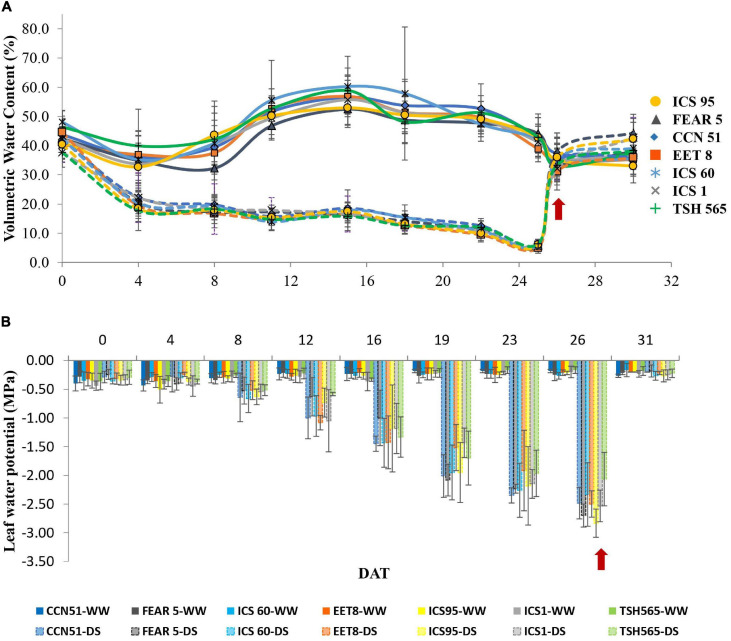
Variation in the **(A)** soil volumetric water content and **(B)** predawn water potential of cacao clones under different water states. WW, well-watered (solid lines); DS, water deficit (dashed lines) conditions. Values are the mean ± SD (*n* = 6). The arrows indicate the exact day of rewatering after 26 days of DS.

For the establishment of the experiment in the glasshouse, the seedlings were planted in black plastic bags that contained 5 kg (10 L) of silty loam soil (pH 6.5). Considering the results of the soil analysis, each plant was fertilized with 5 mL of Agroplus^®^ (Fundases) and 5 g of 15-15-15 (N-P-K) Nutrimon^®^ (Monómeros Colombo Venezolanos S.A) per liter of water. This fertilization scheme was applied twice after grafting to ensure the mineral requirements of the plants for optimal growth and development. From the time of sowing to the application of water stress treatment, all plants were irrigated to field capacity. During the evaluation period, the maximum and minimum temperatures and relative humidity were registered daily with a HOBO 8 weather station (HBOware^®^) installed at 0.50 m above the ground (Supplementary Figure 1A). The mean vapor-pressure deficit (VPD) (Supplementary Figure 1B) was also calculated according to the method proposed by Allen (2006) and dos Santos et al. (2013). The treatments were distributed in a split-plot arrangement under a randomized complete block design, with three replications; the two water states assessed were placed in the main plots: water deficit stress (DS) or field capacity (WW) plants, and the seven clones (Table 1) were placed in the subplots. For the WW treatment, seedlings were well watered to maintain an optimal soil volumetric water content (VWC) of 45% during the evaluation period (Figure 1A). Consequently, the VWC was registered manually at 20 cm depth in the soil of each plant using a FieldScout TDR-300 Moisture Meter (Spectrum Technologies^®^). Under field capacity, the predawn foliar water potential (Ψ_leaf_) remained between −0.2 and −0.4 MPa according to a previously reported Ψ_leaf_ value for well-irrigated cacao plants (Figure 1B; De Almeida et al., 2016). In the DS treatment, irrigation was suspended for 26 consecutive days until Ψ_leaf_ reached values between −3.0 and −3.5 MPa (Rada et al., 2005; dos Santos et al., 2014) moment in which the VWC reached 6% (Figures 1A,B), and the plants showed critical wilting (e.g., leaf senescence and floral bud abortion). According to the Ψ_leaf_ and VWC, day 26 after treatment (D26) was established as the maximum water deficit stress point (Almeida et al., 2002; García Lozano and Moreno Fonseca, 2016). At the end of D26, all plants were irrigated to field capacity for recovery, reaching 45% of the VWC the day after. Recovery measurements were realized during the following 4 days after rehydration (DAR).

### Physiological Parameters Measurements

#### Leaf Water Potential

The Ψ_leaf_ value was measured pre-dawn from 5:00 to 6:30 am in 3 or 4 completely expanded leaves from the top to the bottom of six plants per treatment (*n* = 6) and for each clone (Figure 1B), and it was measured with a Schölander pressure chamber (PMS Model 615, Fresno, CA, United States).

#### Gas Exchange and Intrinsic Water Use Efficiency

The parameters net photosynthesis (A), stomatic conductance (g*_*s*_*) and intercellular CO_2_ (C*_*i*_*) were registered from 9:00 am to 12 m in 3 or 4 completely expanded leaves from top to bottom of nine plants per treatment (*n* = 9) and per clone using a LI-6400XT Portable Photosynthesis System measurement system (LI-COR Biosciences Inc. NE, United States) with an ambient CO_2_ concentration (C_*a*_) of 400 μmoles m^–2^ s^–1^ (De Almeida et al., 2016) and a photosynthetic photon flux density (PFD) of 500 μmoles m^–2^ s^–1^ according to the response of photosynthesis to light for each clone (data not shown). The intrinsic water use efficiency (WUE*_*i*_*) was calculated with A and g_*s*_ data (A/g_*s*_).

#### Chlorophyll Fluorescence Parameters

The maximum quantum yield of PSII (F_*v*_/F_*m*_) was measured in dark-adapted leaves for 45 minutes and the effective quantum yield of PSII (Y[II]) was measured during a repetitive saturating actinic light pulse equivalent to the ambient light (Tounekti et al., 2018), on the same leaves where photosynthesis was recorded (*n* = 9). For this, the chlorophyll molecules were excited for 0.80 s with 1,500 μmoles m^–2^ s^–1^ actinic light. Measurements were realized from 4:00 to 7:00 pm, using a Junior-PAM modulated fluorometer (Walz^®^, Effeltrich, Germany) and the parameters were calculated with WinControl-3 software (Heinz Walz GmbH Inc., Effeltrich, Germany) (Issa et al., 2018; González-González et al., 2020).

#### Relative Leaf Water Content (RWC) and Electrolyte Leakage

The RWC leaf was determined following the protocol proposed by De Almeida et al. (2016) and according to the equation proposed by Slatyer and Shmueli (1967):

$$\text{RWC}\left(\%\right)=\left[\frac{\mathrm{Fresh}\,\mathrm{weight}-\mathrm{dry}\,\mathrm{weight}}{\mathrm{turgid}\,\mathrm{weight}-\mathrm{dry}\,\mathrm{weight}}\right]\times 100$$

The leaf fresh weight, turgid weight and dry weight were determined in leaf pieces of 25 cm^2^ of 21 plants per treatment. The turgid weight was determined after hydrating the pieces with distilled water for 24 hours, and the dry weight was then recorded after drying the pieces in a 70°C oven to a constant weight.

The electrolyte leakage (EL) was determined using the protocol proposed by Souza et al. (2014). For this, ten 6-mm-diameter leaf discs were placed in 15 mL tubes with 10 mL of deionized water at 25°C under constant agitation. The electrical conductivity (EC) was determined with a conductometer (HI 9835 Hanna^®^ - ICT, SL, Bogota, Colombia) after 6 h of incubation and after heating the same sample at 90°C for 2 h. EL was expressed as a percentage (%) according to the equation proposed initially by Stuart (1939):

$$\mathrm{EL}\left(\%\right)=\left[\frac{\text{EC}}{\text{ECmax}}\right]\times 100$$

where, EL is the percentage of lost electrolytes, EC is the EC measured at 6 h, and EC*max* is the EC measured after heating the sample at 90°C for 2 h.

### Gene Expression Profiling

Among the seven clones evaluated, three clones were selected as representatives (see below) of the different responses to DS observed through the physiological variables evaluated. Samples of mature foliar tissue from three plants of each clone were taken on day 0 (control), day 23 and day 26 after DS, once physiological measurements completed (noon) and immediately frozen in liquid nitrogen for total RNA extraction.

#### Identification of Cacao Antioxidant System Orthologous Genes in the Cacao Genome for RT-qPCR Primer Design

A search for genes associated with the water stress response (drought or waterlogging) and oxidative stress (antioxidant system) was performed for model plants in the literature and in sequence databases. Then, the selected CDSs (coding sequences, Table 2) from model plants were used as a query to search orthologous genes in the cacao genome hub database (Argout et al., 2017) using the reciprocal best hit strategy (Ward and Moreno-Hagelsieb, 2014) and running BLAST alignments (Altschul et al., 1990).

**TABLE 2:** Cacao orthologous genes related to the antioxidant system evaluated in this study. The sources of selected genes, expression patterns found in previous studies and primer sequences designed for RT-qPCR analysis are detailed.

Gen	Accession No.	*Tc* Locus name	Forward 5′-3′	Reverse 5′-3′	Length (pb)	Ta	Observed patterns	Species	Author
*SOD3.1*	AAB68035.1	Tc10v2_t014220.1	gtgattcttccaccgtcgtt	caatagcccaacccaaagaa	147	58.5	Overexpression during drought	*Triticum aestivum*	Sheoran et al., 2015
*RBOH F*	AT1G64060.1	Tc03_t028110	acttgccctgtcatttggac	ccattccccactcccttatt	136	58.5	Overexpression during OS	*A. thaliana*	Chaouch et al., 2012; Morales et al., 2016
*GST*	AT1G17170.1	Tc07v2_t011790.1	agccttactttgggggagaa	cacctcttagcccatgcaat	142	57	OS marker	*A. thaliana*	Noctor et al., 2016
*Hsp 17.6*	AT5G12020.1	Tc09_t005320	aaggagggttggcaagttct	ttcaggtggaggcagtttct	124	62.5	OS marker	*A. thaliana*	Noctor et al., 2016
*UGT*	AT4G34131.1	Tc02v2_t008490.1	gggcactgaagaaaaagcag	ccccatagcgatctccatta	160	58.5	Overexpression during OS	*A. thaliana*	Noctor et al., 2016
*NTRC*	AT2G41680.1	Tc05_t017060	ggttcaggtcctgctggata	ctccaggaacaccaccaact	100	57	Overexpression during OS	*A. thaliana*	Kim et al., 2017
*ADH*	AT1G77120.1	Tc08_t012590	gtttttggactgggtgctgt	ttcgacactgcgatcaactc	213	60	Differential expression during waterlogging	*T. cacao*	Bertolde et al., 2014
*PDC*	LOC18596726	Tc06cons_t017450.1	ttgagattcatgatggccctt	ctcctgttgctgtggatattg	150	56	Differential expression during waterlogging	*T. cacao*	Bertolde et al., 2014
*LDH*	LOC18606285	Tc03cons_t024760.1	tggctactccgtggctagct	aagacgccacccctaccaag	150	60	Differential expression during waterlogging	*T. cacao*	Bertolde et al., 2014
*ACPB*	CU536770	Pinheiro et al., 2011	gcagacaagatcagcacaa	aaatcaaagggcacgact	192	52	Housekeeping	*T. cacao*	Pinheiro et al., 2011

*Accession No., indicates the database reference gene identifier; Ta, annealing temperature; *Tc* Locus name, orthologous Gen identifier in Cacao Genome Hub; OS, oxidative stress.*

The best hit of each alignment was selected according to its highest alignment score and lowest E-value and after verifying its coverage length. Once confirmed as orthologs, the cacao CDS was used for qPCR primer design using Primer3Plus Software (Rozen and Skaletsky, 2000) with default parameters. Similarly, primer sequences for three housekeeping genes previously employed in RT-qPCR assays in cacao plants (Pinheiro et al., 2011) were modified to be used as normalizers of the relative expression analysis. Quality control was carried out to confirm the absence of dimer formation, Tm and primer specificity with the Sequence Manipulation Suite (Stothard, 2000) and Primer Quest^®^ (IDT, IA, United States). Finally, a total of nine primer pairs were designed for the respective cacao orthologous genes and three pairs were designed for the housekeeping genes (Table 2).

#### RNA Extraction and cDNA Synthesis

As mentioned above, all harvested leaf samples were maintained in liquid nitrogen and stored at −80°C until RNA extraction. For RNA extraction, an in-house protocol was optimized based on the protocol of Chang et al. (1993), with some modifications as described in Supplementary Material. The RNA concentration and quality were assessed using a 2100 Bioanalyzer (Agilent Technologies^®^, CA, United States). High RNA yield (∼500 ng/μl) with an RNA integrity number (RIN) higher than 7 was obtained after optimization of the protocol. Reverse transcription was performed with 1 μg of total RNA using M-MuLV Reverse Transcriptase (New England Biolabs, MA, United States) following the manufacturer’s instructions and using oligo d(T) 18 primers.

#### Relative Expression Analysis Using RT-qPCR

The RT-qPCR relative expression analysis of each target gene was carried out in triplicate using 3 biological replicates per condition (day 0, day 23, and day 26 after DS). All amplifications included negative template controls run in triplicate. RT-qPCR was performed using a volume of each cDNA equivalent to 5 ng of reverse-transcribed total RNA and 0.25 μM of each primer pair in 15 μl of final reaction volume using Luna Universal qPCR Master Mix^®^ (New England Biolabs, MA, United States). Amplification assays were performed in a Quant Studio 3 Real-Time PCR System (Applied Biosystems, CA, United States). The specificity of all amplifications was verified using melting curves and the threshold cycle values (Ct) of the evaluated and housekeeping genes were registered to calculate the changes in relative expression using the 2^–ΔΔCt^ method (Pfaffl, 2001). Previously, standard curves (Supplementary Figure 2) were obtained for each target gene and amplification efficiencies were calculated according to the criteria suggested by Pfaffl (2004). The expression analyses were normalized with the *ACPB* housekeeping gene, which was the most stable across our conditions and datasets and among all housekeeping genes evaluated. (Supplementary Figure 3). Detailed protocol as well as raw data can be consulted in Supplementary Material.

### Statistical Analysis

Analyses of variance (ANOVA) were performed to determine the effect of the clones and treatments on the physiological variables registered and whether there were significant differences in the relative gene expression of selected genes between clones and days after suspension of irrigation. Subsequently, a mean comparison analysis was performed to assess statistically significant differences through LSD and Tukey’s HSD *a posteriori* tests (*P <* 0.05). The analyses were carried out using R software (R Studio Version 1.1.463) (R Core Team, 2019).

## Results

### Physiological Responses

#### Microclimatic Conditions During Measurements

The air temperature in the glasshouse during the experiment ranged between 17 and 35°C, with a mean value of 26°C, and the average relative humidity was 93% (Supplementary Figure 1A). Furthermore, the VPD ranged between 0.7 and 2.2 kPa, with a mean value of 1.3 kPa (Supplementary Figure 1B).

#### Leaf Water Potential

The Ψ_leaf_ value was significantly different between water states at D26 and between DS plants (Figure 1B). In the WW clones, the Ψ_leaf_ value remained between −0.25 and −0.16 MPa, while in clones under DS, a significant reduction was observed to an approximate value of −3.0 MPa at D26 (Table 3) when the VWC reached 6% (Figure 1A). At D26, not all clones showed similar effect on their Ψ_leaf_, and the clone that showed the highest Ψ_leaf_ was THS 565 (−2.06 MPa) while the clone with the lowest Ψ_leaf_ was ICS95 (−2.83 MPa). Interestingly, at 4 DAR, all clones previously submitted to DS showed similar Ψ_leaf_ values to their respective control (WW) clones (between −0.183 and −0.266 MPa) (Table 3). Therefore, despite experiencing severe water deficit stress of nearly one month without a water supply, all cacao clones were able to recover after rewatering, showing that they were able to tolerate this level of stress.

**TABLE 3:** Leaf water potential (Ψ_leaf_) of the seven cacao clones under different water states.

Clone	Ψ_leaf_ (MPa)		
	
	Water states	D26	DAR
CCN51	WW	−0.1833 ± 0.016 a	−0.266 ± 0.016 a
	DS	−2.48 ± 0.16 cd	−0.200 ± 0.0 a
EET8	WW	−0.2125 ± 0.038 a	−0.200 ± 0.0 a
	DS	−2.50 ± 0.13 cd	−0.216 ± 0.017 a
FEAR5	WW	−0.25 ± 0.06 a	−0.216 ± 0.02 a
	DS	−2.70 ± 0.11 cd	−0.183 ± 0.06 a
ICS1	WW	−0.20 ± 0.06 a	−0.233 ± 0.033 a
	DS	−2.53 ± 0.16 cd	−0.250 ± 0.050 a
ICS60	WW	−0.26 ± 0.03 a	−0.166 ± 0.066 a
	DS	−2.33 ± 0.32 bc	−0.266 ± 0.044 a
ICS95	WW	−0.22 ± 0.02 a	−0.200 ± 0.0 a
	DS	−2.83 ± 0.14 d	−0.266 ± 0.060 a
TSH565	WW	−0.17 ± 0.03 a	−0.183 ± 0.044 a
	DS	−2.07 ± 0.04 b	−0.223 ± 0.060 a

*Values are mean (*n* = 6). Significance (*P* < 0.05) differences after two-way ANOVA (clone x treatment) and LSD *post hoc* test results are denoted by different letters.*

*WW, well-watered, DS, water deficit stress; D26, maximum water deficit stress of 26 days; and DAR, recovery after 4 days of rehydration after DS.*

#### Relative Leaf Water Content and Membrane Permeability Determination

The RWC maintained high values in all WW clones (between 86.36 and 91.33%). At D26, the FEAR5, ICS60, ICS95 and TSH565 clones experienced a significant decrease in RWC (Table 4), with FEAR5 showing the highest water loss (80%). The other stressed clones seemed to be able to maintain RWC values similar to their cognate control plants, with EET8 and CCN51 showing the highest values (87%), with significant differences with respect to the other clones in DS (Table 4).

**TABLE 4:** Changes in the leaf relative water content (RWC) and electrolyte linkage (EL) at D26 in the seven cacao clones.

Clone	Water states	RWC	EL
	
		(%)	(%)
CCN51	WW	89.853 ± 0.88 abc	16.060 ± 1.44 b
	DS	87.450 ± 0.54 abcd	17.350 ± 1.38 b
EET8	WW	86.357 ± 1.97 abcde	15.280 ± 1.40 b
	DS	87.223 ± 1.89 abcd	16.670 ± 0.0 b
FEAR5	WW	89.760 ± 1.07 abc	16.380 ± 1.14 b
	DS	80.093 ± 1.53 e	17.780 ± 1.12 b
ICS1	WW	90.210 ± 1.6 ab	16.563 ± 1.62 b
	DS	83.780 ± 0.54 bcde	23.163 ± 2.16 a
ICS60	WW	91.333 ± 1.29 a	16.557 ± 1.76 b
	DS	83.143 ± 2.79 cde	23.460 ± 3.79 a
ICS95	WW	90.747 ± 1.57 a	16.193 ± 1.91 b
	DS	81.713 ± 4.28 de	19.487 ± 2.25 ab
TSH565	WW	89.923 ± 1.17 ab	15.083 ± 0.80 b
	DS	82.332 ± 3.21 de	16.313 ± 0.94 b

*Values are mean (*n* = 6). Significance differences (*P* < 0.05) after two-way ANOVA (clone x treatment) and LSD *post hoc* test results are denoted by different letters.*

In contrast, EL did not show significant differences between WW and DS in most of the clones, showing a value of 16% on average under DS, similar to all WW plants, except for the ICS1 and ICS60 clones, for which significant increases in EL upon DS were observed, reaching 23.1% and 23.4%, respectively (Table 4).

#### Leaf Gas Exchange

As expected, DS affected leaf gas exchange, significantly reducing A and g*_*s*_*, while C*_*i*_* was increased significantly in all the clones evaluated (Table 5). Under WW treatment, the A values ranged from 3.4 to 5.5 μmoles of CO_2_ m^–2^s^–1^, while under DS, the A values were almost completely inhibited (98% on average) at D26, reaching negative values and ranging between −0.09 and 0.086 μmoles of CO_2_ m^–2^s^–1^, without significant differences between clones. The negative values are indicative of net CO_2_ production in the light (i.e., photorespiration), which is also supported by the higher C*_*i*_* concentration found under DS. Interestingly, despite reaching A values close to 0 μmoles of CO_2_ m^–2^s^–1^ at DS, the ICS1 and EET8 clones did not show negative values, even at D26 (Table 5). As mentioned above, C*_*i*_* increased significantly under DS compared to WW plants as the values rose from 264 to 278 μmol CO_2_ mol^–1^ to 356–447 μmol CO_2_ mol^–1^, accounting for a 45% increase in average due to DS. The highest C*_*i*_* values (447 and 416 μmol CO_2_ mol^–1^) corresponded to ICS95 and FEAR5, for which the increase was 69.1 and 54.2%, respectively, and the lowest values were found for the clones CCN51 and ICS1 (356.8 and 358 μmol CO_2_ mol^–1^), reflecting an increase of approximately 30%. Notably, significant differences were found between the clones presenting the lowest values and those showing the highest values of C*_*i*_* (Table 5).

**TABLE 5:** Changes in gas exchange parameters of the seven cacao clones under different water states.

Clone	Water states		A	g*_s_*	WUE*_*i*_*	C*_*i*_*
		
			(μmol CO_2_ m^–2^s^–1^)	(mol de H_2_O m^–2^s^–1^)	(μmol CO_2_ mol H_2_O^–1^)	(μmol CO2 mol air^–1^)
CCN51	D26	WW	5.525 ± 0.19 a	0.089 ± 0.005 a	62.71 ± 2.7 a	270.50 ± 4.3 d
		DS	−0.009 ± 0.14 d	0.008 ± 0.002 d	−46.27 ± 25.2 d	356.80 ± 8.6 c
	DAR	WW	6.590 ± 0.51 a	0.094 ± 0.010 ab	70.106 ± 3.0 a	256.33 ± 3.8 a
		DS	4.011 ± 0.58 defg	0.052 ± 0.008 cde	77.134 ± 0.9 a	256.00 ± 1.5 a
EET8	D26	WW	5.293 ± 0.56 ab	0.088 ± 0.006 ab	60.15 ± 4.3 a	274.50 ± 7.9 d
		DS	0.086 ± 0.08 d	0.024 ± 0.011 d	7.150 ± 5.0 bc	379.17 ± 11.9 bc
	DAR	WW	6.031 ± 0.72 ab	0.104 ± 0.006 a	57.99 ± 6.6 a	265.83 ± 11.1 a
		R	3.933 ± 0.44 efg	0.051 ± 0.006 cde	77.117 ± 4.0 a	253.17 ± 6.6 a
FEAR5	D26	WW	3.457 ± 0.47 c	0.057 ± 0.007 c	60.67 ± 3.4 a	278.67 ± 4.2 d
		DS	−0.210 ± 0.15 d	0.010 ± 0.001 d	−33.26 ± 14.7 cd	429.67 ± 27.5 a
	DAR	WW	5.001 ± 0.87 abcdef	0.075 ± 0.013 abcde	66.68 ± 5.0 a	258.50 ± 7.4 a
		DS	4.826 ± 0.42 bcdef	0.070 ± 0.013 abcde	68.942 ± 3.4 a	264.33 ± 4.8 a
ICS1	D26	WW	4.655 ± 0.90 abc	0.074 ± 0.014 abc	62.58 ± 2.0 a	276.17 ± 5.1 d
		DS	0.167 ± 0.15 d	0.011 ± 0.001 d	9.08 ± 13.9 b	358.00 ± 19.5 c
	DAR	WW	5.680 ± 0.56 abcd	0.091 ± 0.020 ab	62.417 ± 6.6 a	261.00 ± 10.1 a
		DS	3.748 ± 0.27 efg	0.052 ± 0.004 cde	72.076 ± 2.0 a	263.00 ± 3.6 a
ICS60	D26	WW	4.205 ± 0.71 bc	0.067 ± 0.010 bc	62.65 ± 4.7 a	273.83 ± 8.0 d
		DS	−0.039 ± 0.09 d	0.009 ± 0.002 d	−26.38 ± 17.5 bcd	416.33 ± 28.2 ab
	DAR	WW	5.341 ± 0.78 abcde	0.080 ± 0.013 abcd	66.762 ± 6.5 a	259.33 ± 10.3 a
		DS	3.446 ± 0.49 fg	0.047 ± 0.006 de	73.319 ± 5.1 a	265.33 ± 10.3 a
ICS95	D26	WW	4.460 ± 0.51 abc	0.068 ± 0.010 bc	69.34 ± 3.8 a	264.33 ± 4.9 d
		DS	−0.612 ± 0.34 d	0.012 ± 0.001 d	−49.42 ± 23.7 d	447.00 ± 30.1 a
	DAR	WW	6.038 ± 0.51 ab	0.087 ± 0.012 abc	69.402 ± 6.8 a	252.67 ± 9.6 a
		DS	3.053 ± 0.72 g	0.042 ± 0.009 e	72.69 ± 3.5 a	263.67 ± 6.6 a
TSH565	D26	WW	4.155 ± 0.72 bc	0.060 ± 0.008 c	67.12 ± 4.0 a	270.00 ± 7.8 d
		DS	−0.016 ± 0.12 d	0.010 ± 0.004 d	−22.86 ± 30 bcd	379.00 ± 13.9 bc
	DAR	WW	5.860 ± 0.74 abc	0.087 ± 0.013 abc	67.356 ± 1.9 a	245.67 ± 15.7 a
		DS	4.138 ± 0.44 cdefg	0.057 ± 0.008 bcde	72.596 ± 4.5 a	260.00 ± 7.3 a

*A, photosynthetic rate; g*_*s*_*, stomatal conductance; WUE*_*i*_*, water use efficiency; C*_*i*_*, intercellular CO_2_; WW, well-watered; DS, water deficit stress; D26, maximum water deficit stress of 26 days; DAR, recovery after 4 days of rehydration after DS.*

*Values are mean (*n* = 9). Significance differences (*P* < 0.05) after two−way ANOVA (clone x treatment) and LSD *post hoc* test results are denoted by different letters.*

Furthermore, a significant reduction in g*_*s*_* (80% on average) was observed upon DS treatment in most of the clones compared to their respective WW controls. WW g*_*s*_* values varied from 0.057 to 0.089 mol of H_2_O m^–2^s^–1^, while under DS, the g*_*s*_* values ranged from 0.024 to 0.008 mol of H_2_O m^–2^s^1^.

Moreover, the WUE_*i*_ was also reduced meaningfully under DS in most of the clones and reached negative values, as observed for A. Under WW conditions, the WUE_*i*_ varied between 60 and 69 μmoles of CO_2_ mol H_2_O^–1^, whereas under DS, the values ranged from 7.150 to −49.42 μmoles of CO_2_ mol H_2_O^–1^. Importantly, under DS, WUE_*i*_ was significantly higher for clones ICS1 and EET8 than for the other cacao clones that showed negative WUE_i_ (Table 5).

Overall, the A and g*_*s*_* values of DS plants recovered gradually 4 DAR, reaching an average 50% of WW plant values, while C*_*i*_* was restored to levels similar to those found for WW control plants (Table 5). Interestingly, WUE*_*i*_* reached WW values at 4 DAR for all clones, without significant differences between clones and treatments.

#### Chlorophyll Fluorescence Parameters

At D26, F*_*v*_*/F*_*m*_* decreased significantly in clones under DS compared to WW clones, except for the EET8 clone, for which no significant differences were found. The reduction was in the range of 18 to 44%, with F_*v*_/F_*m*_ values between 0.424 and 0.662 upon DS, compared with values of approximately 0.750 on average for all WW clones. ICS95 and ICS60 showed the highest reduction (44 and 41%, respectively), and the lowest F_*v*_/F_*m*_ values (0.424 and 0.460, respectively) were significantly different from EET8 and CCN51 (Table 6). Concerning Y[II], no significant differences were observed between treatments for EET8, CCN51, ICS1, and TSH565 at the maximum level of stress (D26), whereas under DS, ICS95, ICS60 and FEAR5 experienced a significant Y[II] reduction of 32, 30, and 24%, respectively.

**TABLE 6:** Changes in the maximum quantum yield of PSII (F_*v*_/F_*m*_) and effective quantum yield of PSII (Y (II)) of cacao clones under different water states.

Clone	Water states		F_*v*_/F_*m*_	Y (II)
CCN51	D26	WW	0.767 ± 0.007 a	0.447 ± 0.012 a
		DS	0.613 ± 0.05 cd	0.397 ± 0.033 abc
	DAR	WW	0.743 ± 0.013 abc	0.489 ± 0.014 ab
		DS	0.685 ± 0.031 cde	0.405 ± 0.016 e
EET8	D26	WW	0.756 ± 0.021 ab	0.434 ± 0.005 ab
		DS	0.622 ± 0.042 bcd	0.382 ± 0.054 abcd
	DAR	WW	0.757 ± 0.023 a	0.441 ± 0.010 cde
		DS	0.665 ± 0.025 de	0.435 ± 0.008 cde
FEAR5	D26	WW	0.740 ± 0.010 abc	0.450 ± 0.016 a
		DS	0.556 ± 0.047 de	0.341 ± 0.049 bcd
	DAR	WW	0.757 ± 0.011 a	0.452 ± 0.012 bcd
		DS	0.696 ± 0.035 abcde	0.408 ± 0.026 de
ICS1	D26	WW	0.760 ± 0.007 a	0.454 ± 0.023 a
		DS	0.502 ± 0.007 de	0.384 ± 0.033 abcd
	DAR	WW	0.7485 ± 0.017 abc	0.476 ± 0.016 abc
		DS	0.700 ± 0.032 abcde	0.436 ± 0.016 cde
ICS60	D26	WW	0.791 ± 0.016 a	0.432 ± 0.008 ab
		DS	0.460 ± 0.062 e	0.301 ± 0.053 cd
	DAR	WW	0.755 ± 0.003 ab	0.438 ± 0.020 cde
		DS	0.689 ± 0.025 bcde	0.429 ± 0.023 de
ICS95	D26	WW	0.758 ± 0.009 a	0.436 ± 0.005 ab
		DS	0.424 ± 0.108 e	0.295 ± 0.074 d
	DAR	WW	0.752 ± 0.021 abc	0.442 ± 0.012 cde
		DS	0.7130 ± 0.009 abcd	0.438 ± 0.019 cde
TSH565	D26	WW	0.752 ± 0.011 ab	0.472 ± 0.012 a
		DS	0.523 ± 0.047 de	0.441 ± 0.016 ab
	DAR	WW	0.721 ± 0.021 abcd	0.500 ± 0.014 a
		DS	0.634 ± 0.037 e	0.427 ± 0.008 de

*WW, well-watered; DS, water deficit stress; D26, maximum water deficit stress of 26 days; DAR, recovery after 4 days of rehydration after DS.*

*Values are mean (*n* = 9). Significance differences (*P* < 0.05) after two-way ANOVA (clone x treatment) and LSD *post hoc* test results are denoted by different letters.*

Regarding the effect of rehydration on the recovery of photosynthesis, all clones, including those that were more affected by DS, were able to recover values similar to their control (WW) clones without significant differences between them at 4 DAR (Table 6). In general, the response of all evaluated clones to the DS treatment seemed similar, although important differences were observed that allowed us to identify some clones with a better capacity to cope with the stress, as mentioned below.

Among the differences in the response to DS observed between clones, interestingly, the EET8 clone did not show a decrease in either the F*_*v*_*/F*_*m*_* parameter or Y[II], while the ICS60 clone was one of the most affected clones in these two parameters together with a significant increase in EL, as stated above. The impact of these three parameters, particularly F*_*v*_*/F*_*m*_*, could reflect photoinhibition by oxidation, assessing the existence of this condition to be relevant in *T. cacao*. Therefore, to determine both the extent of this probable oxidative stress induced under DS and whether different cacao clones responded differently regarding the activation of their antioxidant system, gene expression profiling was carried out on three selected cacao clones: the two most contrasting clones with regard to their photosynthetic and chlorophyll fluorescence parameters, as well as the EL parameters (EET8 and ICS60), together with the TSH565 clone, which maintained the highest water potential under DS.

### Drought and Oxidative Stress-Related Gene Expression Profiling

The genes used to evaluate the activation of an oxidative stress response under DS in *T. cacao* (Table 2) were grouped into three categories according to the function to which they have been associated according to the literature: (1) markers of oxidative stress (Noctor, 2006), (2) crosstalk between water deficit and oxidative stress (Noctor, 2006; Chaouch et al., 2012; Sheoran et al., 2015; Morales et al., 2016; Kim et al., 2017), and (3) a possible switch to fermentative metabolism (Bertolde et al., 2014). Furthermore, to observe the progressive change in the expression patterns of these genes in response to the severity of water deficit, these genes were evaluated before, near to the end and at the end or maximum level of DS, which corresponded to days 0, 23, and 26, respectively.

#### Oxidative Stress Markers GST and HSP

**FIGURE 2:**
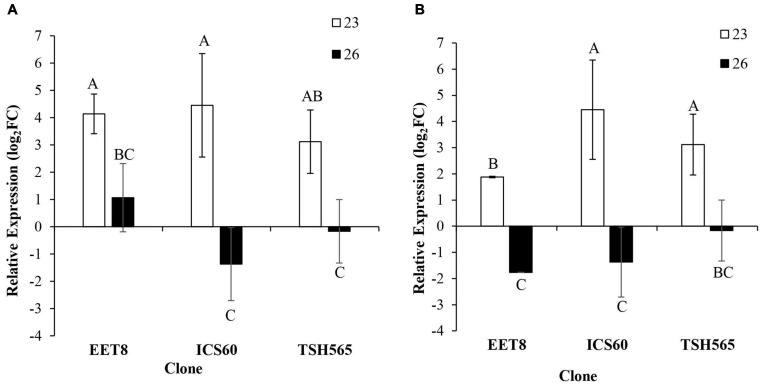
Effect of water deficit on the transcriptional expression of oxidative stress markers. **(A)** *GST*. **(B)** *Hsp17.6*. Bars represent the averages of the induction factor (log2-fold change) ± standard deviation (*n* = 3) relative to the control (day 0). The letters indicate statistically significant differences between days 23 and 26 of water deficit (*P* < 0.05).

Significant differences in the expression patterns of these two oxidative stress markers were found after water deprivation in all three clones evaluated (Figure 2). Interestingly, strong upregulation was observed at D23 in all clones, and it was significantly reduced or undetectable at the maximum stress level (D26). Although no significant differences were found between clones for *GST* expression profiles, at D26, EET8 was the only clone to maintain induction of both *GST* and *HSP* genes compared to both ICS60 and TSH565 clones, which showed a downregulation pattern at the maximum level of DS (D26). In contrast, for the *HSP* gene, the upregulation observed at D23 was significantly higher in ICS60 and TSH565 clones than in EET8 clones. Although there were few differences in the expression profiles between clones, the strong induction of both oxidative stress markers observed at D23 before the maximum level of stress suggests that DS triggered oxidative stress in all cacao clones

#### Crosstalk Between Water Deficit and Oxidative Stress

**FIGURE 3:**
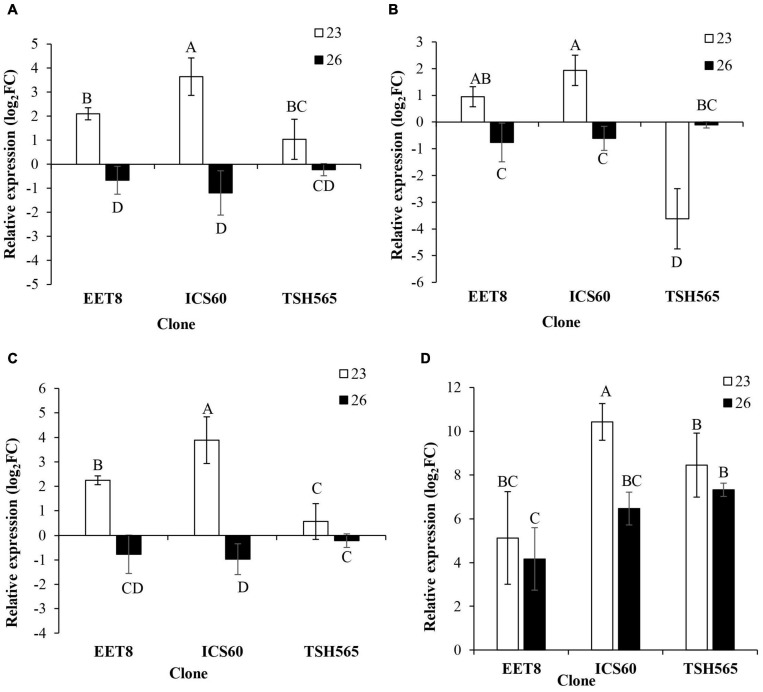
Effect of water deficit on the transcriptional expression of genes encoding antioxidant enzymes. **(A)** *RBOHF*. **(B)** *NTRC*. **(C)** *UDPGT*. **(D)** *SOD*. Bars represent the averages of the induction factor (log2-fold change) ± standard deviation (*n* = 3) relative to the control (day 0). The letters indicate statistically significant differences between days 23 and 26 of water deficit (*P <* 0.05).

Furthermore, genes encoding enzymes of the antioxidant system (*RBOHF*, *NTRC*, *UDPGT*, and *SOD*) were analyzed according to their expression profile in response to DS in cacao. In general, all genes showed a similar pattern to that found for *GST* and *HSP*, i.e., a significant induction at D23 in all clones (Figure 3), except for TSH565, which presented a downregulation of *NTRC* expression at this point (Figure 3B). Interestingly, *RBOHF*, *UDPGT*, and *SOD* showed greater induction in the ICS60 clone than in the other clones. Nonetheless, the *UDPGT* induction levels were significantly higher in ICS60 followed by EET8, and the lowest induction was observed for TSH565 (Figure 3C). Finally, as observed for *GST* and *HSP*, most genes experienced a reversion of this upregulation in all clones at D26, except for *SOD* in TSH565 and EET8 clones in which this ROS transcript maintained an upregulated state at this point, while in ICS60, it showed a significant reduction between D23 and D26 (Figure 3D).

#### Possible Switch to Fermentative Metabolism

**FIGURE 4:**
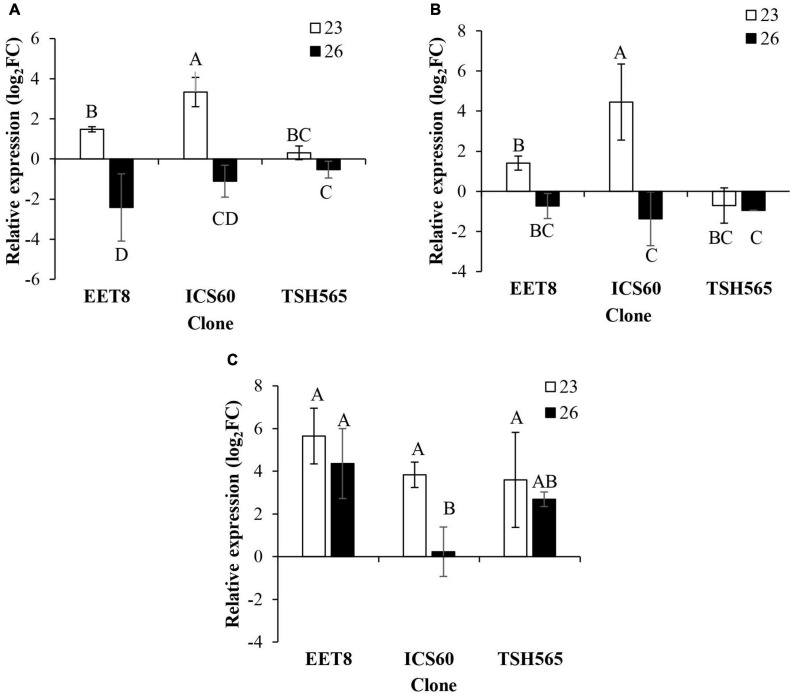
Effect of water deficit on the transcriptional expression of genes encoding fermentative metabolism enzymes. **(A)** *LDH*. **(B)** *PDC*. **(C)** *ADH*. Bars represent the averages of the induction factor (log2-fold change) ± standard deviation (*n* = 3) relative to the control (day 0). The letters indicate statistically significant differences between days 23 and 26 of water deficit (*P* < 0.05).

Interestingly, the expression patterns of fermentative enzymes coding transcripts showed similar general behavior to that observed for antioxidant enzymes, i.e., they all presented a significant upregulation at D23 in the three clones except for the *PDC* transcript in TSH565, which was downregulated (Figure 4). Induction patterns at D23 presented differences between clones for *LDH* and *PDC* transcripts, and in both cases, a significantly higher level of induction was found for ICS60. In contrast, no differences in induction levels were found for *ADH* transcripts between the three clones. Finally, as observed for most of the antioxidant enzymes, reversion of this upregulation at D26 was presented for *LDH* and *PDC* transcripts in all clones, whereas *ADH* induction seemed maintained in all clones, although a significant reduction was observed in ICS60 (Figure 4C).

## Discussion

### Physiological Responses of Cacao Clones to Drought

This research aimed to characterize and compare the physiological responses of seven commercial cacao clones. Our results show some clear differences between clones, reflecting different levels of tolerance among them, although all clones were able to recover from a pronounced water deficit stress of 26 days long. One key variable that is typically recorded in plants under drought conditions and which is directly associated with tolerance to DS is Ψ_leaf_ because it indicates the plant water status and the plant’s ability to uptake soil water and avoid water loss (dehydration) (Bray, 1997; Rodríguez et al., 2017). In this study, Ψ_leaf_ decreased in all cacao clones to values below −2.3 MPa at the maximum level of DS (D26). However, the TSH565 clone showed the highest Ψ_leaf_ value (−2.07 MPa), indicating that it was less affected than the rest of the clones (Table 3), which suggests a higher capacity of this clone to maintain water homeostasis in the leaf tissues and cell turgor under severe water deficit. Balasimha et al. (1991) noted that drought-tolerant cacao genotypes were able to maintain less negative Ψ_leaf_ during the driest months of the year. Therefore, this ability shown in the TSH565 clone indicates that this genotype is tolerant to water deficit stress (Balasimha et al., 1991, 2013; Medina and Laliberte, 2017). Moreover, this reduction in Ψ_leaf_ under limiting conditions is related to the low availability of water in the soil since the VWC of the soil dropped gradually to 6% after suspending the plant irrigation (Figure 1A). García Lozano and Moreno Fonseca (2016) reported that at 10% VWC, the soil lacks available water for plants, and Medina and Laliberte (2017) proposed that in cacao, severe stress occurs below water potential values of −1.76 MPa. Our results are consistent with previous studies carried out in cacao and report that the reduction in Ψ_leaf_ to values below −1.3 MPa (García Lozano and Moreno Fonseca, 2016) is a direct consequence of the reduction in the soil VWC caused by periods of drought (Alban et al., 2016; De Almeida et al., 2016; García Lozano and Moreno Fonseca, 2016; Lahive et al., 2019). Indeed, upon rewatering at 4 DAR, the cacao clones restored Ψ_leaf_ to values close to those of WW plants (Figure 1B).

In addition, it has been observed that reduced water supply impacts cacao growth and development through a reduction in the leaf area, which leads to lower net assimilation, stomatal conductance, and yield, being this reduction of leaf area considered one of the earliest acclimation mechanisms to withstand drought (Ayegboyin and Akinrinde, 2016). Impact of DS in cacao leaf area was addressed in a different study in which only ICS60, TSH565 and EET8 clones were monitored: a significant reduction in the leaf area upon DS was observed, without differences between clones. However, TSH565 had the lesser reduction (47%) with respect to its control (WW) plants, while EET8 and ICS60 clones showed a reduction in leaf area of 72% (Osorio-Zambrano, 2021). Thus, TSH565 response could be related with the ability of this clone to cope with water deficit stress by maintaining higher water potential, as has been observed here.

In previous research, stomatal sensitivity for regulating water loss has also been proposed as an important physiological trait of adaptation to drought stress, as it may reflect a better ability to cope with DS during the resulting acclimation response of the plant and represents the main mechanism to efficiently control water equilibrium and avoid water loss by transpiration (Almeida and Valle, 2007; Apshara et al., 2013). However, because of the strong impact of the evaporative demand of the atmosphere (FAO, 2006; García Lozano and Moreno Fonseca, 2016; Merilo et al., 2018; Lahive et al., 2019), during the experiment time course, microclimatic variables were monitored to evaluate their influence on physiological parameters. Although VPD tended to increase with temperature accordingly, the VPD mean value (1.3 kPa) indicated that evaporative demand did not contribute to a decrease in g*_*s*_* or A in the plants, since the normal stomatal closure values in cacao are close to 1.8 KPa as reported previously (García Lozano and Moreno Fonseca, 2016). Indeed, in *T. cacao* plants, increases in VPD caused a linear decrease in g*_*s*_*; however, A only decreased at VPD values up to 2.0 kPa (Balasimha et al., 1991; Almeida and Valle, 2007; Baligar et al., 2008; García Lozano and Moreno Fonseca, 2016; Lahive et al., 2019), a threshold that was not reached in our experimental time course.

Furthermore, the reduction in g*_*s*_* under DS, as a direct consequence of stomatal closure and despite being an acclimation response of plants (Osakabe et al., 2014), is also considered a trait related to tolerance, as it is part of the avoidance response mechanism to reduce water loss by transpiration under water deficit and thus to maintain RWC, which prevents tissue damage (Gupta et al., 2020). In the current study, the reduction in g*_*s*_* by almost 80% in all cacao clones evaluated (Table 5) suggests a stomatal-sensitive response for the regulation of water loss by transpiration, with the consequent reduction in A due to the reduction in CO_2_ uptake. Despite these similarities between clones, EET8 showed the highest g*_*s*_* at D26 under DS (Table 5), which may have influenced the maintenance of higher CO_2_ assimilation, as reflected both by a positive A compared to the other clones as well as a higher F_*v*_/F_*m*_ value reflecting no effect of PSII photochemical activity under DS. In contrast, the ICS60 clone exhibited the highest reduction in g*_*s*_* together with a negative A value under DS, indicative of photorespiration and no net carbon assimilation, clearly showing a reduced performance of this clone under DS and therefore a lesser stress tolerance (Apshara et al., 2013; Balasimha et al., 2013).

This stomatal-driven decrease in A and g*_*s*_* near and below zero values at D26 in cacao clones is consistent with previous studies on the response of cacao to DS (Ismail et al., 1992; Rada et al., 2005; Araque et al., 2012; Acheampong et al., 2013; De Almeida et al., 2016). In addition, the negative values were linked to the high rate of photorespiration found in cacao due to the high point of CO_2_ compensation exhibited and attributed to a possible photoprotective role (Avila-Lovera et al., 2016). These studies were carried out in greenhouses and fields with young and adult plants, and drought treatments were imposed and caused by the dry season, respectively. Furthermore, Joly and Hahn (1989) mentioned that under severe water deficit stress, CO_2_ assimilation drops notably, reaching zero due to turgor loss and becoming negative when respiration overtakes photosynthesis.

Finally, our findings are in accordance with a previous study where different trinitarian cacao hybrids (TSH clones) were evaluated (Antwi et al., 1994), and the most tolerant clone to DS showed little or no change in g*_*s*_* and E while the most susceptible clones to DS presented earlier declines in g*_*s*_*. Among the evaluated clones, TSH919 was proposed as the most tolerant to DS based on its higher Ψ_leaf_ under stress conditions, thus corroborating the use of this parameter as key for the DS tolerance ranking of cacao clones. Cacao adult trees growing in the field displayed a similar behavior, with a significant reduction in Ψ_*leaf*,_ A, and g_*s*_ upon DS, however, it has been observed that this reduction could be less pronounced than in a glasshouse, due to a higher soil water content during the dry season. Under this scenario, the root system could delay the sensing of water deficit, although a smaller decrease in Ψ_leaf_ during dry season is considered a drought-tolerance trait (Balasimha et al., 1991; Rada et al., 2005; Araque et al., 2012; Avila-Lovera et al., 2016; De Almeida et al., 2016).

As mentioned above, the observed stomatal closure allowed it to keep the RWC above 80% in all clones at D26 under DS, thus maintaining leaf turgor and preventing tissue damage, which is a known mechanism of DS avoidance. Similar responses have been found in other cacao clones exposed to water deficit without a reduction in RWC (Almeida et al., 2002; Rada et al., 2005; Almeida and Valle, 2007; De Almeida et al., 2016). This ability has also been attributed to an osmotic adjustment mechanism, which contributes to maintaining leaf hydration and cell turgor during DS (Medina and Laliberte, 2017). A similar behavior was observed in coffee cultivars submitted to drought stress, whose capacity to recover from dehydration was related to osmotic adjustment triggered by the accumulation of proline and other compatible osmolytes (Tounekti et al., 2018). Additionally, this mechanism may be involved in the maintenance of membrane structural integrity and prevention of oxidative damage by scavenging ROS, which induce oxidative degradation of the lipid bilayer, thus causing electrolyte leakage. Therefore, its activation under DS may prevent essential solutes from leaking out (Villar-Salvador et al., 2004; Valentovic et al., 2006; Parkash and Singh, 2020). Complementary biochemical analyses aiming at quantifying the accumulation of compatible solutes or the activation of their cognate biosynthetic genes are needed to corroborate this probable osmoregulation response (Almeida and Valle, 2007; Araque et al., 2012).

Equally important, the WUE*_*i*_*, a variable that relates A and g*_*s*_* (Daymond et al., 2011; Lahive et al., 2019), indicates the water uptake by roots with minimal stomatal opening and thus less water loss through transpiration. It is also an indicator of the maintenance of atmospheric CO_2_ assimilation and thus photosynthesis. Therefore, it is also considered a key variable often associated with drought tolerance when comparing genotypes (Alban et al., 2016). In the current study, WUE*_*i*_* was markedly reduced under DS in all cacao clones compared to the WW plants. However, at D26, the ICS1 and EET8 clones presented higher WUE*_*i*_*, which suggests that these two clones have developed mechanisms to optimize the balance between ensuring a minimal photosynthetic rate and reducing water loss through transpiration, both dependent on g*_*s*_* reduction. This capacity of a genotype to maintain carbon assimilation and photosynthesis under reduced water availability is reflected in a high WUE_*i*_ and indicative of a higher stress tolerance (Daymond et al., 2011). Conversely, the reduction in WUE*_*i*_* has been attributed to severe stress status, possible damage to the photosynthetic apparatus or oxidative stress occurrence (Lahive et al., 2019). Interestingly, variation (i.e., increase or decrease) in WUE_*i*_ in response to drought seems to be a genotype-dependent trait, which has been observed previously and was also the case in our study (Araque et al., 2012; Almeida et al., 2014; Avila-Lovera et al., 2016; Lahive et al., 2019).

In addition to and related to the relationship between carbon fixation and stomatal closure, the C_*i*_ parameter also appears to be a good indicator of the rate of effective CO_2_ consumption under stomatal conductance limitation: accordingly, under DS, EET8 presented lower C_*i*_ values than ICS60, indicating a higher CO_2_ consumption rate, allowed by a higher g*_*s*_*, and reflected in the higher photosynthetic performance (A) observed for the EET8 clone compared to ICS60.

Altogether, the above results clearly support that the cacao response to water deficit is strongly governed by stomatal conductance, as the first limiting factor of photosynthetic performance under DS, with the consequent drop in A, as has been previously reported for other cacao genotypes (De Almeida et al., 2016). This is likely to be an early and efficient mechanism encountered in several other tropical perennial crops like mango, coffee or avocado (Damour et al., 2009; Ramalho et al., 2018; Tounekti et al., 2018; Martínez-Ferri et al., 2019), as well as temperate deciduous trees such as peach or apple (Liu et al., 2012; Jiménez et al., 2013). However, our results related to chlorophyll fluorescence showed also a reduction in both F_*v*_/F_*m*_ and Y[II] values upon DS in clones that exhibited a lesser tolerance based on water status or gas exchange parameters (i.e., ICS60 and ICS95). In contrast, stressed EET8 clones showed F_*v*_/F_*m*_ and Y[II] values similar to those obtained in their cognate WW controls. F_*v*_/F_*m*_ reduction has been related to a photoinhibition phenomenon, with the concomitant reduction in Y[II] and increase in C_*i*_. The cacao response to DS may therefore have two limiting phases depending on the genotype: one initial and stomatal-controlled phase, resulting in a substantial reduction in A and C*_*i*_* as g*_*s*_* decreased, followed by a second and strongly photosynthetically limited phase that caused an increase of C*_*i*_* while F*_*v*_*/F*_*m*_* and Y[II] decreased as an indication of the onset of non-reversible photoinhibition and photosynthetic apparatus damage. These two sequential phases have been described in the response to severe DS in other woody and perennial crops (Brodribb, 1996; Damour et al., 2009; Tounekti et al., 2018). A similar response was found in previous studies carried out with other cacao clones submitted to DS, either in glasshouse or field conditions, and with different light intensity regimes (i.e., sunlight or shade light): in all cases, a reduction in F*_*v*_*/F*_*m*_* to values between 0.2 and 0.7 and a concomitant reduction in Y[II] were observed (Araque et al., 2012; Acheampong et al., 2013; Balasimha et al., 2013; Alban et al., 2016; Kumar and Jegadeeswari, 2019). According to Baker (2008), plants without chronic photoinhibition present F*_*v*_*/F*_*m*_* values in the range of 0.71 to 0.83 and oxidative damage is observed when basal fluorescence increases and F*_*v*_*/F*_*m*_* values are below 0.6, as observed in most of the clones under DS in our present study. The rapid loss of PSII photochemical efficiency, as a result of water deficit, has been attributed to possible damage to the PSII light-harvesting complex caused by stress-induced oxidative burst, increased leaf chlorosis or decreased root carbohydrate concentrations, with subsequent plant death (Bertolde et al., 2012, 2014; Sharma et al., 2020). However, all evaluated clones were able to recover rapidly, reaching values of F*_*v*_*/F*_*m*_* at 4 DAR either similar or higher than WW plants, indicating that the observed drop in photosystem efficiency and possible damage were reversible, even after maximum DS. This response could be related to oxidative stress, photoinhibition and metabolic changes that together trigger plant photoprotective mechanisms as well as water homeostasis and energetic metabolism maintenance rather than irreversible damage (Bhattacharjee and Saha, 2014). Efficient PSII repair mechanisms, such as through the activation of D1 protein turnover or size adjustment of the photosynthetic antenna by the degradation of chlorophyll-binding proteins, as a preventive mechanism of photoinhibition, and activation of the antioxidant system, are considered key tolerance mechanisms of plants to DS (Knopf and Adam, 2018) because they can lead to a complete recovery of plant photosynthesis.

Furthermore, rapid inhibition of photochemistry with irreversible damage to the photosynthetic apparatus occurs in plants that cannot recover predrought levels of A and show visual signs of leaf damage or senescence after continued drought (Brodribb, 1996). In our case, the overall values of A, F_*v*_/F_*m*_ and Y[II], which are marked by a fast recovery of the predrought state and observed for all cacao clones, could strongly suggest the activation of the abovementioned efficient damage repair mechanisms.

Finally, EL is an indicator associated with membrane integrity and stability that has also been linked to drought tolerance (Bajji et al., 2002; Souza et al., 2014). This physiological parameter has also been related to osmotic adjustment because it can prevent damage to the membrane and the consequent loss of electrolytes and contributes to cellular turgor maintenance. Specifically, all cacao clones showed similar responses and were able to maintain cell turgor and consequently membrane integrity without electrolyte leakage through leaf mesophyll cells, which could be related to the osmotic adjustment mechanism (Villar-Salvador et al., 2004; Parkash and Singh, 2020). In the case of ICS60 and ICS1 clones, the slight increase observed in EL could indicate possible DS-induced membrane damage, which may have occurred by lipid peroxidation due to ROS formation and oxidative stress response, a phenomenon also found in cacao seedlings subjected to Cu toxicity (Souza et al., 2014). Similarly, several parameters indicated the ICS60 clone appears to be more affected by DS and therefore more susceptible than the other clones.

Overall, the above-discussed results concerning the physiological response of cacao clones to DS suggest that the exposure time to water stress by suspension of irrigation for 26 days represented severe water deficit stress for all clones evaluated. Although all clones were able to recover quickly (at 4 DAR), the results provided insights indicating that the clones presented differences in their responses related to their capacity to tolerate DS, at least under our experimental conditions. Thus, our results indicate that the EET8 clone was the most performant under DS, whereas ICS60 was seemingly the less tolerant. These differences could reflect changes in acclimation responses, which in turn could be either genetically or epigenetically dependent (Shinozaki et al., 2017).

On the other hand, as photoinhibition mechanisms were observed under DS, which suggests secondary oxidative stress induction, a preliminary approach to the activation of the antioxidant system at the transcriptional level was carried out in these clones to complement these findings.

Besides, considering that grafting practice is frequently used to integrate traits of interest between the rootstock and the scion, which in cacao is mainly destinated to confer disease resistance (Ribeiro et al., 2016), and that the rootstock could alter scion responses to various biotic and abiotic factors (Jiménez et al., 2013; Serra et al., 2014; Martínez-Ferri et al., 2019; Kapazoglou et al., 2021), it is possible that the responses observed here could have been influenced by the root-scion interaction. This may have contributed either to certain variability within each clone, due to the genetic variability of the sexually propagated rootstock, or to a more similar behavior between clones, due to the use of the same rootstock. Although the aim of this study was to compare the physiological response of grafted clonal materials, which is the most widespread propagation practice, addressing more specifically the effect of root/scion interaction on cacao response or tolerance to water deficit becomes relevant for future studies, as shown by some researches in progress (Ayegboyin, 2012; Medina and Laliberte, 2017; Lahive et al., 2019).

### Oxidative Stress Response Gene Expression Profile

Only the EET8, ICS60, and TSH565 clones and leaf organ responses were evaluated in this section, since they showed most of the differences in behavior according to photosynthetic parameters, as well as in water potential. Interestingly, our gene expression profiling results appeared to confirm that photochemical (nonstomatal limited) reactions were affected and that a typical oxidative stress response occurred in cacao upon DS. Indeed, gene activation related to oxidative stress-related genes was seemingly directed to protect leaf cells because the expression of canonical oxidative stress repair marker genes *GST* and *HSP* showed high induction in the three clones evaluated at D23, which likely had a much earlier induction. This finding could be related to an effort of cacao plants to prevent or attenuate the negative effects of ROS increase triggered by the DS imposed. This activation has been reported as an early response induced in plants to protect protein structures and lipids against possible damage caused by ROS and tends to accumulate as a consequence of photorespiration (Chen et al., 2019; Laxa et al., 2019; Sharma et al., 2019).

As stated above, this kind of damage was also evidenced by the low values of F*_*v*_*/F*_*m*_*, which likely affected A, and the drop in g*_*s*_*, with both responses evidenced during DS. *GST* is a key enzyme in ROS detoxification due to its conjugation with glutathione (Dixon and Edwards, 2005; Ding et al., 2017; Gullner et al., 2018), its relationship with the enzyme glutathione peroxidase and the detoxification of lipidic hydroperoxides (Gullner et al., 2018). The induction of *GST* expression has been linked as an early and key mechanism in Arabidopsis, wheat and tobacco for ROS detoxification triggered by abiotic stress (Ezaki et al., 2004; Noctor et al., 2016; Chen et al., 2019). In addition, *Hsp17.6* belongs to the small heat shock protein family and is mainly found in plants and linked to ROS tolerance because it protects enzymes related to the maintenance of metabolic pathways encoded by either housekeeping or stress-related genes (Kotak et al., 2007; Park and Seo, 2015; Mishra et al., 2018). Therefore, the upregulation of *Hsp17.6* has been described as a molecular signature indicating the severity of oxidative stress (Al-Whaibi, 2011; Noctor et al., 2016; Mishra et al., 2018). Although these oxidative stress markers were induced under DS in all three clones evaluated, the EET8 clone presented the lowest induction fold change in both genes, suggesting that the oxidative burst or adverse intracellular damages to repair were reduced in this clone compared to the others, accordingly with its respective physiological behavior, as discussed previously.

Similarly, *RBOHF* showed a significant induction at D23 in all three evaluated clones, interestingly not maintained at D26, when the maximum level of DS was reached. This expression pattern (i.e., a strong induction followed by a downregulation) may indicate that the transcriptional activation of this pathway in *T. cacao* could have happened earlier, even before D23, when the water status-related physiological parameters started to decrease and the related stress was perceived systemically by the plant. Moreover, this early upregulation could also be related to the physiological regulation of the stress response since the *RBOHF* protein exerts regulatory functions on ABA, the master mediator of the systemic response to DS (Kwak et al., 2006; Osakabe et al., 2014). Among its several plant regulatory functions, ABA influences guard cell turgor and therefore regulates stomatal closure (Kwak et al., 2006; Mittler and Blumwald, 2015), which is essential to reducing water loss, which was observed in all the cacao clones evaluated (Table 5). The downregulation of *RBOHF* at D26 was observed after a previous activation, and it is a common pattern of expression found for many stress-response genes and does not necessarily reflect either the cognate protein level or its activity. Nevertheless, such a pattern more likely obeys either a temporally limited function associated with its signaling and regulatory roles, which is necessary for metabolic energy saving under stress conditions, or to possible relays of activation that appear to occur in a cascade as part of the complex gene regulatory network controlling the water-deficit stress response in plants (Shinozaki et al., 2017).

Interestingly, the ICS60 clone, which showed a higher *RBOHF* induction, also showed significantly higher induction of *SOD* at D23 than the TSH565 and EET8 clones. However, it should be noted that these clones also exhibited high induction of *SOD*. In any case, the coordinated induction of *RBOHF* and *SOD* suggests that a compensatory mechanism may be activated to counteract ROS accumulation in response to DS at least at the transcriptional level (Laxa et al., 2019), thus orchestrating gene induction by retrograde signaling as part of a systemic acquired acclimation response (Mittler and Blumwald, 2015) necessary for the tolerance and recovery of the evaluated cacao clones.

Concerning *NTRC* expression profiling, although downregulation was observed at D23 for the TSH565 clone, which is inconsistent with the induction observed for ICS60 and EET8, downregulation was the common pattern for all three clones at D26. *NTRC* appears to follow a similar expression pattern to *HSP* and *RBOHF* (i.e., induction followed by repression at the maximum level of stress), suggesting probable coregulation as part of the common gene regulatory network controlling this systemic response to DS. Indeed, thioredoxins (*NTRCs*) are disulfide reductases that regulate the redox state of other proteins and lipid peroxides. Therefore, their function has been related to the maintenance of metabolic pathways occurring in chloroplasts, such as photosynthesis, because of stress conditions (Vieira Dos Santos and Rey, 2006; Chaves et al., 2009). The *NTRC* protein family has been shown to protect chloroplasts from photooxidative damage generated by light stress and drought, both in rice (Perez-Ruiz, 2006) and Arabidopsis (Serrato et al., 2004; Toivola et al., 2013; Kim et al., 2017). Furthermore, there is evidence indicating that thioredoxins could act as regulators of ROS scavenging and as components of signaling pathways in the antioxidant system response of plants (Kim et al., 2017).

Similarly, *UDPGT* followed a similar profile, i.e., upregulated at D23 and downregulated at D26 in all clones. As *UDPGT* belongs to a family of enzymes responsible for glycosylation of regulators of plants, flavonoids, terpenoids and steroids (Sun et al., 2013), its activation is necessary to maintain the bioactivity of enzymatic cofactors and regulators of plants, including ABA, during stress conditions (Sun et al., 2013; Rehman et al., 2018). As reported by Li et al. (2017), the overexpression of *UGT79B2/B3* increases anthocyanin accumulation, improving antioxidant activity under salinity stress, low temperatures and drought in Arabidopsis. In this study, we evaluated the ortholog in *T. cacao* of the *A. thaliana* AT4G34131.1 (*UGT73B3)* gene (Table 2). Rehman et al. (2018) proposed that this gene has a role in drought tolerance and oxidative stress response. Accordingly, the registered induction of *UDPGT* suggests that it could have similar functional roles, thus helping the plants address both kinds of stresses.

Ethanol synthesis and its accumulation have been reported as indicators of stress in plants, such as water deficit stress, thus affecting cellular energy via oxidative phosphorylation (Kelsey and Westlind, 2017). These results may occur due to aerobic respiration depletion, either by limiting the O_2_ supply or by mitochondrial damage (Manter and Kelsey, 2008; Schmidt et al., 2018). Under an adequate O_2_ supply, tissues metabolize pyruvate by oxidative phosphorylation, while with low O_2_ concentrations (hypoxia) or complete depletion (anoxia), the cytoplasmic pH drops, increasing the activity of the two fermentation enzymes (pyruvate decarboxylase (*PDC)* and alcohol dehydrogenase (*ADH)*) responsible for ethanol accumulation in the cytoplasm of cells. This ethanol allows the regeneration of NAD^+^ needed for glycolysis to continue producing the energy (ATP). In addition, it contributes to maintaining membrane integrity and its minimal functioning and stabilizing cellular pH, thereby avoiding lethal acidosis until aerobic respiration resumes (Kelsey and Westlind, 2017). Here, *PDC, ADH*, and *LDH* transcripts were evaluated to assess the other effects of the DS treatment on energy metabolism.

In this respect, the *LDH* gene, which encodes a key enzyme involved in fermentative metabolism, was also upregulated at D23 and downregulated at D26 in the three clones. This pattern of expression suggests that the physiological stress caused by DS possibly triggered hypoxia in the cacao plants either by limiting oxygen supply or by accumulated damage in the mitochondria. Consequently, energy production by activation of fermentative metabolism may be part of the acclimation strategy upon DS in cacao. When hypoxia occurs, the predominant pathway is alcohol fermentation, where pyruvate is converted to acetaldehyde by pyruvate decarboxylase and then to ethanol by alcohol dehydrogenase (Dolferus et al., 2008). However, in addition to ethanol fermentation, plants also have a lactic acid fermentation pathway, where lactate dehydrogenase converts pyruvate to lactate. The relative activity of both fermentation pathways has often been correlated with tolerance to hypoxia or anoxia (Dolferus et al., 2008). Then, an increase in the levels of the *LDH* transcript, recorded at 23D, suggests that possibly the encoded protein accumulated in an early phase to attend to the hypoxic condition later.

Consistent with the above, the ICS60 and EET8 clones showed induction of the *PDC* gene at D23 and all clones showed upregulation of *ADH* at both D23 and D26. Upregulation of the enzymes PDC and ADH is essential to maintain the generation of ATP, the energy piece required to keep the general metabolism functioning (Felle, 2005; Loreti et al., 2005). Although the *LDH*, *PDC* and *ADH* genes do not seem to follow a fully coordinated expression pattern between clones, it was evident as a general tendency that cacao may have switched its energetic metabolism from oxidative to fermentative as part of its acclimation response to DS, at least at the transcriptional level, which needs further experimental analysis at the protein expression and enzymatic activity level. Most of the leaves in all clones entered senescence at the maximum level of DS (D26) (visual evaluation), which could be a possible explanation for the general downregulation pattern observed at the transcriptional level for some of the fermentative metabolism genes, as well as all genes evaluated, and caused by a drastic change in energetic metabolism, with a probable trade-off between fermentative metabolism and maintenance respiration. However, transcriptional repression of a gene does not necessarily imply a drop in its respective protein level or activity, which could be maintained further. A similar behavior was described in conifers, which showed that all aerial tissues of drought-stressed seedlings accumulated ethanol when the tissues were near death or dying (Manter and Kelsey, 2008).

The expression patterns of fermentative genes observed here upon DS were not previously reported in cacao, although these gene patterns have been observed in *A. thaliana*, mainly in roots and stems (Dolferus et al., 2008; Mithran et al., 2014; Dumont et al., 2018). However, the induction of these genes was reported in cacao plants under waterlogging (Bertolde et al., 2014). These proteins and the different proteins associated with photosynthesis and oxidative stress were proposed as determining factors to distinguish between a tolerant genotype (TSH-792) to waterlogging and a susceptible genotype (TSH-774) (Bertolde et al., 2014).

Most land plants experience mild flooding, but not all species have developed adaptations to grow and survive drastic changes in water availability in their environment (Jackson and Colmer, 2005; Jackson et al., 2009; Bailey-Serres et al., 2012; Herrera, 2013). It appears that *T. cacao* can induce the activation of fermentative pathways mediated by LDH, PDC, and ADH both in water excess and deficit, which in addition to being part of an acclimation response to probable stress-induced hypoxia may also constitute an adaptive plasticity trait to respond to different hydrological regimes naturally occurring in the Amazon rainforest, a seasonally flooded region that is also the central region of origin of *Theobroma* species (Motamayor et al., 2008; Cornejo et al., 2018). Altogether, these findings suggest the need for further integrated genetic, transcriptional, physiological, and biochemical characterizations of cacao germplasm for directing breeding to climate change adaptative traits. In particular, comparisons of these responses between its ten major genetic cluster will provide more insights into the different adaptative and acclimation counterparts of the cacao response and tolerance to DS.

## Concluding Remarks

The results of the current study confirmed that the irrigation suspension period applied in the seven cacao clones led to severe drought stress. As an initial early response to DS, cacao plants exhibited regulation of stomatal closure (stomatal limitation) linked to the stress-avoidance mechanism; then, when DS became severe, a second phase response emerged with the downregulation of photosynthetic efficiency (nonstomatal limitation) as a consequence of oxidative stress and photoinhibition caused by DS. However, all plants recovered quickly (4 DAR), probably by mediating the antioxidant system as well as the efficiency of PSII repair mechanisms and the turnover of chloroplasts and their components, which represent crucial responses for maintaining photosynthesis and metabolism under stress conditions (Yang et al., 2019). Although all stressed cacao clones recovered, EET8 exhibited the highest tolerance, TSH565 exhibited a moderate response and ICS60 exhibited the highest susceptibility. Overall, the physiological response observed suggests that these cacao clones showed high phenotypic plasticity (De Almeida et al., 2019) and different levels of tolerance to drought (Tezara et al., 2020) that may allow them to respond to DS with diverse efficiency and varied responses at different levels. We report for the first time new insights into the cacao response to DS related to the participation of the antioxidant system and its probable role in physiological readjustment, protection, and energetic metabolism maintenance. These results are particularly important for seedling establishment in the field in which the frequency of irrigation can have an impact in production and yield due to the greater susceptibility of seedlings or young trees to dehydration (Almeida and Valle, 2007; Ayegboyin and Akinrinde, 2016; Medina and Laliberte, 2017). Furthermore, improving cacao to obtain varieties more tolerant to future climatic conditions caused by global warming is becoming more and more necessary, this requires research that integrates genetic, physiological, and multiple functional genomics approaches to elucidate the genetic and epigenetic bases of cacao DS responses and tolerance.

## Data Availability Statement

The raw data supporting the conclusions of this article will be made available by the authors, without undue reservation.

## Author Contributions

MO, LR, and WT conceived the project and experimental design. DC designed the gene expression profiling. MO and DC carried out the experiments, collected, analyzed data, and wrote the initial versions of the manuscript. All authors contributed to manuscript revision and improvement, read, and approved the submitted version.

## Conflict of Interest

The authors declare that the research was conducted in the absence of any commercial or financial relationships that could be construed as a potential conflict of interest.

## Publisher’s Note

All claims expressed in this article are solely those of the authors and do not necessarily represent those of their affiliated organizations, or those of the publisher, the editors and the reviewers. Any product that may be evaluated in this article, or claim that may be made by its manufacturer, is not guaranteed or endorsed by the publisher.
