# Supplementary material for: Cacao (Theobroma cacao L.) Response to Water Stress: Physiological Characterization and Antioxidant Gene Expression Profiling in Commercial Clones
Source: Front Plant Sci. 2021 Sep 6;12:700855. doi: 10.3389/fpls.2021.700855 (PMC8450537; doi:10.3389/fpls.2021.700855)
Supplement: Supplementary file 1 [file Data_Sheet_1.pdf]

A

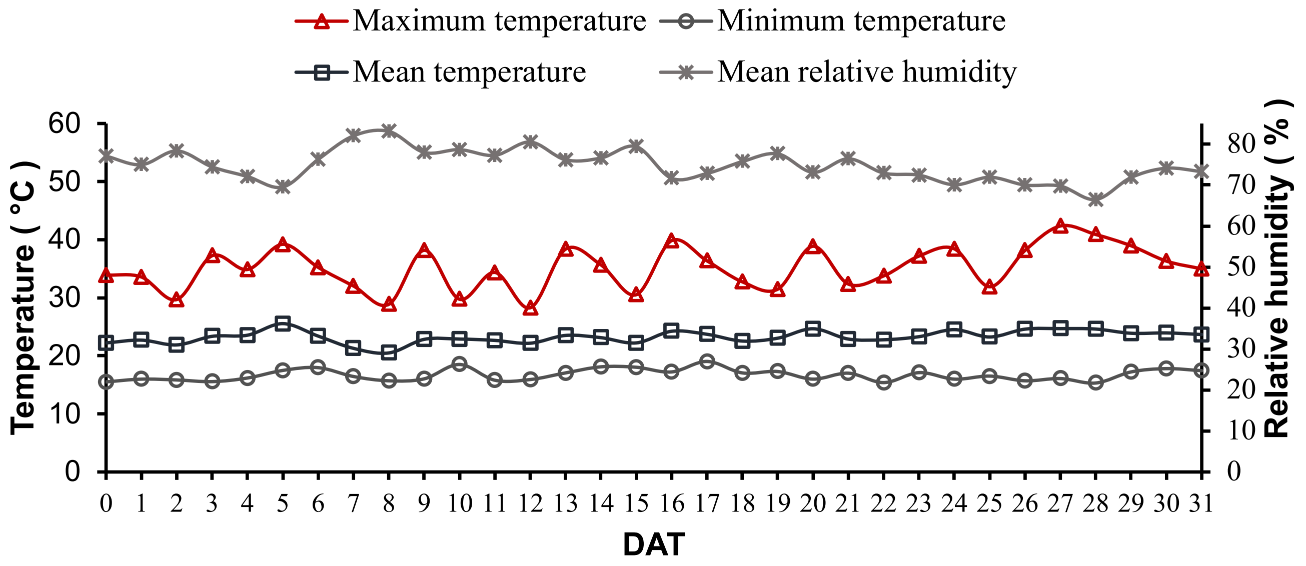

B

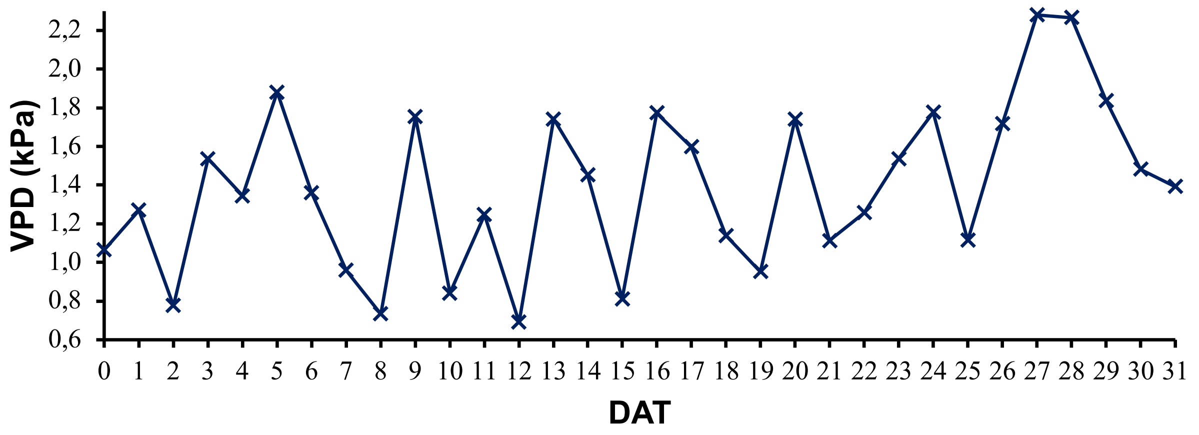

Supplementary figure 1. Variation in the **(A)** daily average temperature and relative humidity conditions and **(B)** vapor pressure deficit (VPD) in the glasshouse during the period of water deficit and recovery.

Supplementary information: Detailed protocol for RNA extraction and RT-qPCR assays.

Total RNA extraction from cacao leaf tissue:

For cacao leaf RNA extraction, an in-house protocol was optimized based on the protocol of Chang et al. (1993) with the following modifications: 200 mg of leaf tissue per sample were ground to powder in liquid nitrogen and transferred to a 2 ml tube previously frozen. Then 1.4 ml of pre heated (65°C) Extraction Buffer [100 mM Tris, pH 8.0; 2 M NaCl; 25 mM EDTA; 2 % CTAB (w/v); 2% PVP-10,000 (w/v); 2 % β-mercaptoethanol (v/v) and 0.05 % spermidine (w/v)] was added and homogenized by vortex. The samples were then incubated in a dry bath at 65°C for 15 minutes, shaking the suspension every 2 minutes. The lysate was then centrifuged for 2 in at room temperature and the supernatant was divided into two tubes of 5Prime Phase Lock Gel (Quantabio, MA, USA) and 700 ul of chloroform:isoamyl alcohol [24:1 (v/v)] was added to each tube, homogenized by inversion, and centrifuged at 12,000 g for 20 minutes at 4°C, the supernatants were transferred to two new 5Prime Phase Lock Gel tubes repeating the previous step. The supernatant recovered from the two tubes was transferred to a 2 ml tube and ¼ volume (~ 250 ul) of 10 M LiCl was added, homogenized by inversion and the RNA was precipitated at 4°C overnight. RNA precipitate was then sedimented by centrifugation at 12,000 g for 20 minutes at 4°C. The supernatant was discarded, and the pellet was resuspended in 500 ul of pre-heated (30° C) Buffer SSTE [1 M NaCl, 0.5 % SDS (w/v), 10 mM Tris-HCl (pH 8.0) and 1 mM EDTA]. The mixture was transferred to a 5Prime Phase Lock Gel tube, then, 500 ul of chloroform:isoamyl alcohol [24:1 (v/v)] were added and mixed by inversion, then centrifuged at 12,000 g for 20 minutes at 4°C and the supernatant was transferred to a 1.5 ml tube. Subsequently, two volumes (~ 1 ml) of 70 % ethanol (v/v) were added and the RNA was precipitated for 2 hours at -80 ° C. RNA precipitate was sedimented by centrifugation at 12,000 g for 20 minutes at 4°C and the supernatant discarded with a micropipette. RNA pellet was spinned again for 1 minute to decant the residual liquid, which was collected again with a micropipette and discarded. The RNA pellet was dried before being resuspended in 50 ul of ultra-pure DEPC Treated water and heated to 37°C in a dry bath for 5 minutes. Immediately, the RNA was transferred to ice and the RNA concentration and quality were assessed using a NanoDrop® 1000 Spectrophotometer (Thermo Fisher Scientific, MA, USA) and a 2100 Bioanalyzer (Agilent Technologies®, CA, USA). High RNA yield (~500 ng / µl) and RNA integrity number (RIN) higher than 7 were obtained after optimization of the protocol.

Checking of DNA contamination:

The absence of DNA contamination in RNA samples was confirmed by conventional PCR using the following primers of the ß-tubulin (ßTUB) gene (CU525140; Tc08cons_t003810.1): Fw Primer: 5´-ATTCCCCCGTCTTCACTTCT-3´, Rev Primer: 5´-TCTGCTCATCAACCTCTTTGG-3´ (custom synthetised at Macrogen, Korea). PCR reactions ere carried out with Gotaq 2X Green Master Mix (Promega, WI, USA), following manufacturer instructions, using each primer at 0.35 uM each and using with 100 ng RNA from each sample as template in a final volume of 25 uL. PCR cycling temperature were: 5 min. at 95°C, followed by 35 cycles of: 30 seg. at 95°C, 30 seg. at 54°C and 30 seg. at 72°C, ending with 7 min. at 72°C. The expected amplicon length was 212 pb, checked in standard agarose gel electrophoresis.

cDNA Synthesis and primer specificity checking:

Reverse transcription (RT) was performed with 1 ug of total RNA using M-MuLV Reverse Transcriptase (New England Biolabs, MA, USA), following the manufacturer instructions, and using oligo d(T)18 primer. The obtained cDNA was stored at –20 °C until qPCR assays were performed. The specificity of the primers pairs of each target gene (custom synthetised at Macrogen, Korea) was evaluated first by conventional PCR (Gotaq 2X Green Master Mix - Promega, WI, USA) using a volume of cDNA equivalent to 10 ng of total RNA and 0.25 each primer, in a final volume of 20 uL. Optimal annealing temperatures were assessed using temperature gradients of 0.5 ºC in a range of 5 to 7 ºC from the theoretical melting temperature of each primer pair, and until obtaining a single amplification product and no evidence of primer dimer formation. The size of the PCR product was confirmed by agarose gel electrophoresis [1.5 % agarose (w/v), Buffer TBE 0.5X, and Hydra Green™ Safe DNA Dye (ACTGene, NJ, USA) following manufacturer instructions.

qPCR standardization

The qPCR Standardisation was performed using MicroAmp™ Optical 8-Tube Strip with Attached Optical Caps, 0.2 ml (Applied Biosystems, CA, USA). qPCRs were run with each pair of primers and standard curves were made using serial dilutions of the cDNA (10.00, 1.00, 0.10 and 0.01 ng equivalents of RNA) in triplicates. Negative template controls and RT- control (using 10 ng of total RNA as template) were also run in triplicate for each primer pair. qPCR was performed using Luna Universal qPCR Master Mix® (New England Biolabs, MA, USA) with 0.25 µM of each primer pair in 15 µl of final reaction volume. Amplification assays were performed in a Quant Studio 3 Real-Time PCR System (Applied Biosystems, CA, USA). The slopes of the standard curves and the linear regression coefficients (R^2) were calculated to estimate the linear detection range and the amplification efficiencies (E) (Supplementary Fig. 1). Amplification efficiency was calculated according to Pfaffl (2004). Furthermore, specificity of amplification was checked in all cases by generating Melting Curves (see Melting Curves-Sheet in the Raw data file).

The housekeeping gene selected as reference gene for the normalization of the differential expression analyses was ACPB based on its higher expression stability accross the different sampling days of the DS treatment.and genotypes *(T. cacao* clones) according to Eissa et al., (2016). (Supplementary Fig. 2 and housekeeping stability-Sheet in the Raw data excel file)

Reference:

Eissa, N., Hussein, H., Wang, H., Rabbi, M. F., Bernstein, C. N., & Ghia, J. E. (2016). Stability of reference genes for messenger RNA quantification by real-time PCR in mouse dextran sodium sulfate experimental colitis. PLoS ONE, 11(5), 1–23. https://doi.org/10.1371/journal.pone.0156289

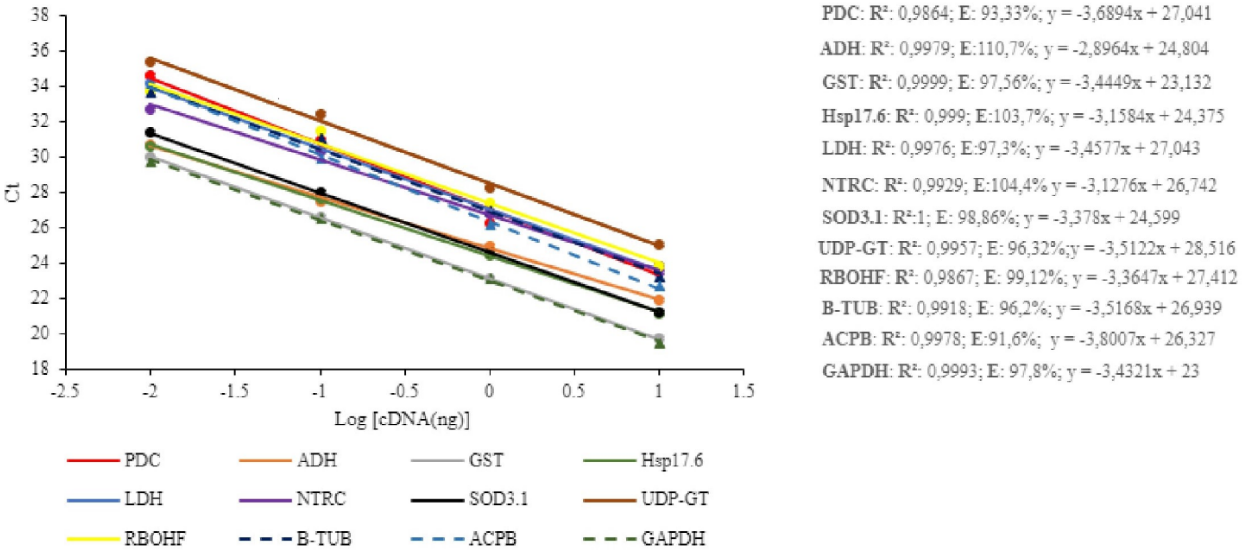

Supplementary Figure 2. **Standard curves of all RT-qPCR targeted genes.** The plotted values correspond to the averages resulting from the serial dilutions 10.00, 1.00, 0.10 and 0.01 ng of cDNA, (n = 3). For each gene, the values of R^2, amplification efficiency (E) and equation of the linear regression are presented, where Y axe corresponds to the Ct value obtained and X axe corresponds to Log [cDNA] in nanograms.

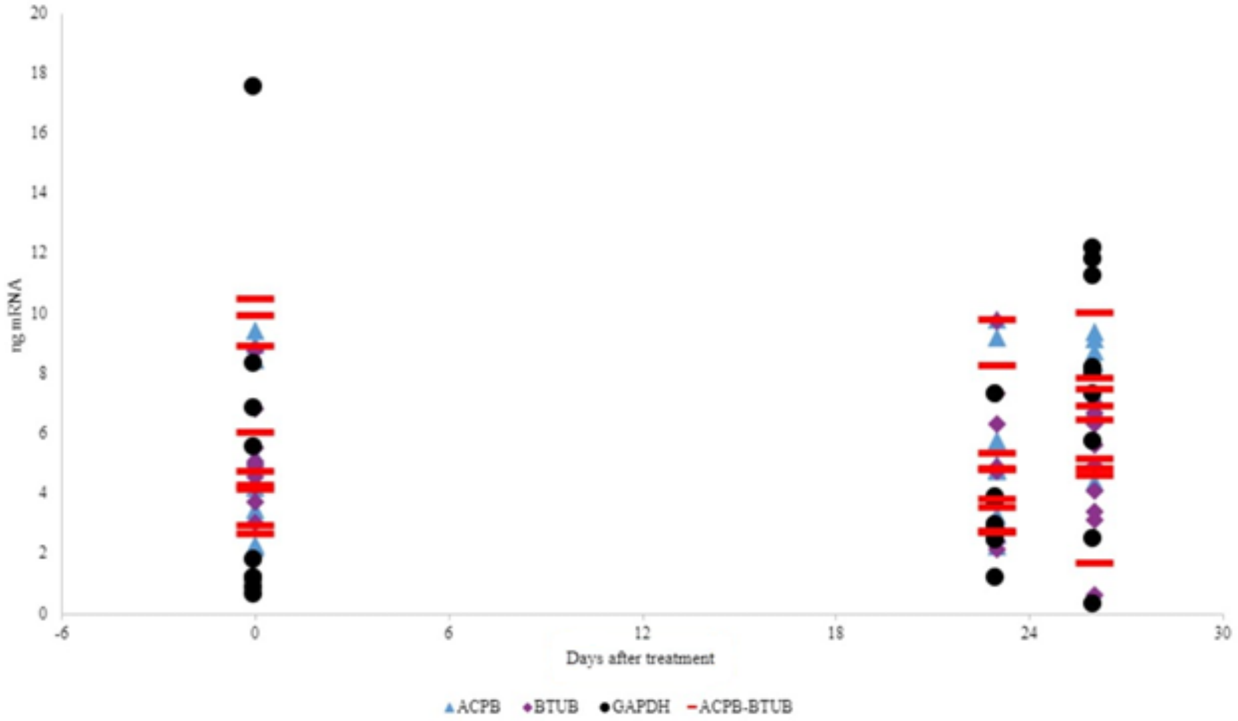

Supplementary Figure 3. **Stability of transcriptional expression of *T.cacao* housekeeping genes upon DS.** Y axis values correspond to absolute expression expressed in equivalent of ng of mRNA of each housekeeping gene evaluated at 0, 23 and 26 days after treatment (DAT) and in the three clones (EET8, TSH565 and ICS60).

Supplementary Table 1. Analysis of Variance (ANOVA) for Ψ_{leaf}

Source	DF	SS	MS	F	P
Clone	6	0.6223	0.1037	1.75	0.1445
Treatment	1	55.5350	55.5350	938.05	0.0000***
Clone*Treatment	6	0.5032	0.0839	1.42	0.2421
Error	29	1.7169	0.0592		
Total	42				

Note: SS are marginal (type III) sums of squares
Grand Mean -1.3533 CV -17.98

Signif. codes: 0 ‘***’ 0.001 ‘**’ 0.01 ‘*’ 0.05 ‘.’ 0.1 ‘ ’ 1

If the ANOVA found a significant overall treatment effect, LSD test (Least Significant Difference, P ≤ 0.05) was carried out to compare means between treatments within clones.
